# Blind Witnesses Quench Quantum Interference without Transfer of Which-Path Information

**DOI:** 10.3390/e22070776

**Published:** 2020-07-16

**Authors:** Craig S. Lent

**Affiliations:** Department of Electrical Engineering and Department of Physics, University of Notre Dame, Notre Dame, IN 46556, USA; lent@nd.edu

**Keywords:** quantum computation, quantum device, quantum information, decoherence, which-path information, entropy

## Abstract

Quantum computation is often limited by environmentally-induced decoherence. We examine the loss of coherence for a two-branch quantum interference device in the presence of multiple witnesses, representing an idealized environment. Interference oscillations are visible in the output as the magnetic flux through the branches is varied. Quantum double-dot witnesses are field-coupled and symmetrically attached to each branch. The global system—device and witnesses—undergoes unitary time evolution with no increase in entropy. Witness states entangle with the device state, but for these blind witnesses, which-path information is not able to be transferred to the quantum state of witnesses—they cannot “see” or make a record of which branch is traversed. The system which-path information leaves no imprint on the environment. Yet, the presence of a multiplicity of witnesses rapidly quenches quantum interference.

## 1. Introduction

Two slit quantum interference is arguably the paradigmatic quantum effect. Maxima and minima analogous to Young’s famous double-slit optical experiment illustrate the importance of the superposition of distinct possible dynamic paths. The vanishing of the interference if one measures which path the particle actually takes is crucial to the mystery. As Feynman puts it, “when we look at the electrons, the distribution of them on the screen is different than when we do not look” [1].

There is now no doubt both that this is true and that it reveals a fundamental feature of the physical law. The recent Bell test experiments have shown that the quantum indeterminacy which resides in a superposition state is a feature of reality and not just one’s knowledge of reality [2,3,4,5]. A measurement does not actually reveal a pre-existing fact (local realism), but rather it forces the physical world, so to speak, to make a choice between physically allowed but distinct possible outcomes.

**Figure 1:**
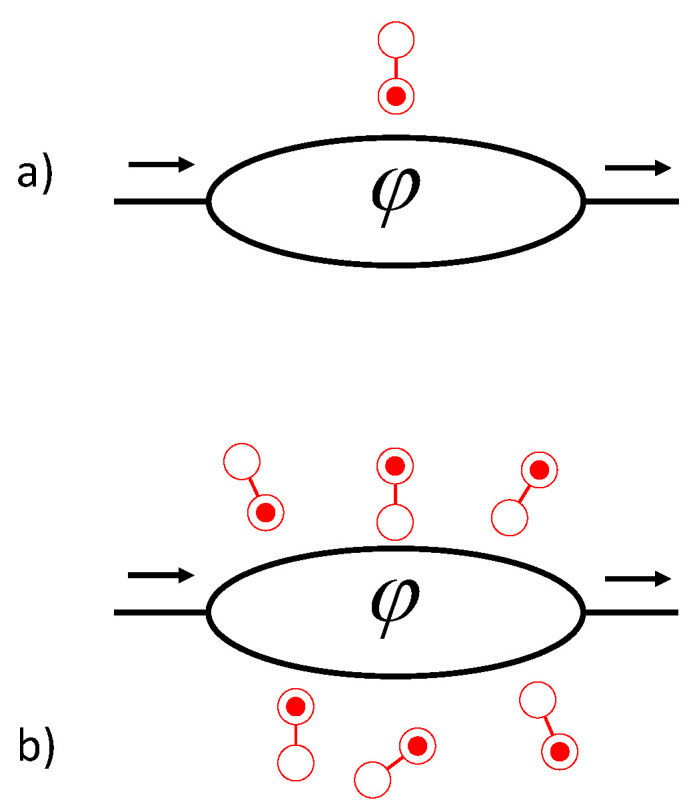
The role of measured ancilla or unmeasured environmental witnesses in a two-branch quantum interference device. (**a**) In an idealized version of the device in [6], measurement of the state of an ancillary double-dot system suppresses coherent oscillations in the output as the enclosed magnetic flux φ is varied. This can be interpreted as the result of the transfer of which-path information from the system to the ancilla when the two become entangled prior to measurement. (**b**) Double-dot systems here represent the many witnesses present in the random environment. Though the witnesses are not measured, the entanglement of the system with the witness degrees of freedom results in the extinction of coherent oscillations in the output. The environment can be said to receive imprinted information about the state of the system.

Quantum computation exploits superposition states of spatially separated systems, so decoherence due to interaction with the environment or a measurement device is important to understand in detail. If a measurement yields unambiguous information about a system’s dynamical path—clear which-path information—then system coherence is lost and there is no interference at all. However, a measured result is subject to noise and finite precision, so loss of interference admits of degrees. The interference pattern can be reduced in visibility without being eliminated entirely. Buks et al. observed electron interference in a two-path GaAs device modulated by a perpendicular magnetic field [6]. A which-path detector composed of a quantum dot and quantum point contact was added to one path. Reduction in the visibility of the interference pattern was correlated with the sensitivity of the detector. Figure 1a illustrates a simplified version of this device in which a quantum double dot acts as an ancilla whose measured state discloses which-path information about an electron traversing the double-branched channel.

Diffraction of C${}_{70}$ matter waves in a vacuum chamber was observed by Hackermuller et al. [7]. Interference fringe visibility was gradually quenched with increasing partial pressure of argon [8]. In this case, there was no measurement of the C${}_{70}$ path, but rather decoherence due to a transfer of which-path information from the C${}_{70}$ to argon atoms, which scatter off the molecule and act as unmeasured witnesses. The random environment entangles with the device system and produces decoherence [9,10].

Wootters and Zurek [11] applied the tools of information theory to analyze the double-slit interference problem for photons, making quantitative the central argument of the Bohr–Einstein debate at the Fifth Solvay Conference. Extending this work, Englert derived an inequality [12] that connects the distinguishability, which characterizes a which-path detector to the interference visibility. The complications of multi-slit interference has been examined by others [13,14]. Broader questions about the nature of the detector, its disturbance on the observed system, and the roles of uncertainty and complementarity have all impacted the discussion. Recently, addressing quantum measurement, Patekar and Hofmann [15] emphasized the need to clearly distinguish the role of system-meter entanglement from the projective measurement of distinguishable meter states.

It is clear that decoherence does not require a measurement. Decoherence is now commonly described in terms of the movement of information, using terms such as “information deposited in the environment” and “environment as witness” [16], “information transfer from the system to the environment” [17], etc. Transfer of which-path information to the environment is certainly sufficient to cause decoherence, but is it a necessary condition?

Quantum Darwinism [18] is based on the observation that certain system information survives to become classical and objective because it leaves a great multiplicity of imprints on the environment. Figure 1b illustrates a random environment interacting with a double-branch interference device. Quantum double dots act as environmental witnesses which, though unmeasured, can record an imprint of the system path information, as in the case of argon atoms scattering off the C${}_{70}$ molecules mentioned above.

However, what if the witnesses *cannot* record the which-path information of the system? We consider just such a case. We construct a simple model for a two-path quantum interference device. Multiple double-dot witnesses are placed symmetrically in ordered arrays on both branches of an interference device; randomness plays no role. Both the device and the witnesses are symmetric with respect to the two paths. The witnesses are further constrained so they are not actually able to sense or record which-path information—the tunneling matrix elements between the witness quantum dots is zero. We therefore call these blind witnesses. They are incapable of receiving an imprint of this information from the system. In such a highly symmetric situation, one might expect that quantum interference would endure, but we show that the presence of these blind witnesses still quenches the interference.

We solve for the unitary dynamics of a wave packet traversing the device and examine the interference pattern at the output as a function of the strength of an applied magnetic field. The witness states are characterized by both their quantum degrees of freedom and their entropy.

Note that this is not the regime of weak quantum measurement—the interaction between the witnesses and the system is not weak, and there is no measurement. Because the time evolution described here is unitary, the decoherence seen is, of course, reversible in principle. No global information is lost and the overall entropy is always zero. Irreversible decoherence would result if either measurement occurred or if the system interacted with a thermal bath.

We consider a model environment composed of an ordered symmetric array of blind witnesses, not because it represents a more realistic description of actual environments, but because it allows us to distinguish the effect of environmental randomness from the barest consequences of a multiplicity of witnesses.

Section 2 describes the model of the quantum interference device in the absence of witnesses, and in Section 3, we solve for the dynamics of the system alone. Section 4 adds in the witnesses and shows the effect they have on the quantum interference. In Section 5, we examine the dynamics of the witnesses themselves to see how their quantum degrees of freedom evolve. The final section discusses how the quenching of quantum interference originates in the multiplicity of even symmetrically placed blind witnesses, without the transfer of which-path information from the system.

## 2. Model System

**Figure 2:**
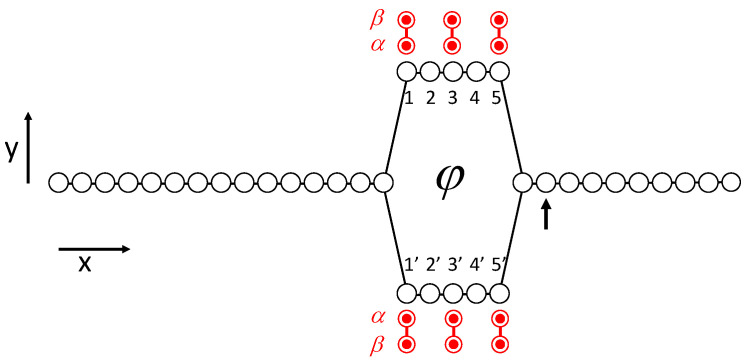
Model quantum interference device geometry. A tight-binding type site model with near-neighbor tunnel coupling represents the interference device. The input lead on the left is connected through upper and lower branches with the output on the right. A perpendicular magnetic field creates a magnetic flux φ through the loop. An input wave packet is injected from the left and emerges on the right. The arrow on the right indicates the output site $j_{out}$. Each witness consists of two dots, labeled α and β, which are field-coupled to sites on the upper and lower branches. The presence of an electron at the adjoining device site raises the energy if the α site of the witness is occupied. The geometry illustrated is the case of 6 witnesses at sites $\left[1,1^{\prime },3,3^{\prime },5,5^{\prime }\right]$. Minimal “blind” witnesses are constrained to always have equal occupancy probabilities for the α and β sites, as illustrated here by the solid (red) circles.

We consider the basic quantum two-path interference device with the geometry shown in Figure 2. We model the device using a tight-binding type Hamiltonian with N=35 discrete sites. A line of sites on the left splits into top and bottom branches and then merges again on the right. (At this point, ignore the double-dot witnesses on the top and bottom branches shown in the figure.) We consider an electron (with charge −e) incident from the input lead on the left and examine the output on the right. Let $|j\rangle$ represent the state with the particle localized on the $j^{th}$ site at position ${\vec{r}}_{j}=\left(x_{j},y_{j}\right)$. Site 1 on the left edge of the input lead is at the origin of the coordinate system. The *x*-coordinates of the sites are integer multiples of a distance *a*, though the magnitude of *a* will finally play no role. The *y*-coordinates are 0 for the input and output leads, and ±a/2 for the top and bottom branches. For clarity in labeling, the vertical and horizontal distant scales in Figure 2 are different. The index *j* runs from 1 to 15 for the input lead, from 16 to 20 for the top branch, 21 to 25 for the bottom branch, and from 26 to 35 for the output lead. The branch sites are additionally (redundantly) labeled 1 through 5 in the top branch and $1^{\prime }$ through $5^{\prime }$ in the bottom branch for convenience in discussing the witnesses below.

A uniform perpendicular magnetic field $\vec{B}$, described by the vector potential $\vec{A}=-By\hat{x}$, produces different quantum phase shifts for the electron traversing top or bottom branches, creating varying amounts of constructive or destructive interference at the output. The magnetic flux through the loop formed by the top and bottom branches is φ. The strength of the applied field can be specified by choosing $\varphi /\varphi_{0}$, where $\varphi_{0}=2\pi \hbar /e$ is the magnetic flux quantum.

The input lead, top and bottom branches, and output lead constitute the interference device (as distinct from the witnesses) and we can write the device Hamiltonian:

(1) $${\hat{H}}_{d}=\sum_{i,j}t_{i,j}|i\rangle \langle j|+t_{i,j}^{*}|j\rangle \langle i|.$$

The hopping matrix element $t_{i,j}$, using the Peierls substitution [19] to account for the magnetic field, is given by

(2) $$t_{i,j}=-\gamma \;e^{-i\frac{e}{\hbar }\int_{{\vec{r}}_{j}}^{{\vec{r}}_{i}}\vec{A}\cdot d\vec{r}}$$

for those sites, *i* and *j*, which are connected as indicated by the lines in Figure 2, and zero otherwise. At the points where the input and output leads connect to the two branches, the magnitude of the connecting matrix element remains γ for simplicity, even though the distance between sites is $a\sqrt{2}$.

## 3. Dynamics without Witnesses

Given an initial state, we find the state at a future time *t* directly using the unitary time evolution operator.

(3) $$|\psi (t)\rangle =e^{-i\frac{{\hat{H}}_{d}}{\hbar }t}|\psi (0)\rangle$$

The natural time scale τ of the motion depends on the the magnitude of the hopping matrix element γ.

(4) τ≡πℏ/γ.

Consider a wave packet initially in the input lead and moving to the right. At time t=0 we set the wave function on the input lead to be

(5) $$\langle j\;|\;\psi (0)\rangle =A\;e^{-{\left(x_{j}-x_{0}\right)}^{2}/\left(2w^{2}\right)}\;e^{ikx_{j}}.$$

Here, we take $x_{0}=5a$, w=2a, and ka=π/2, with *A* chosen for normalization.

**Figure 3:**
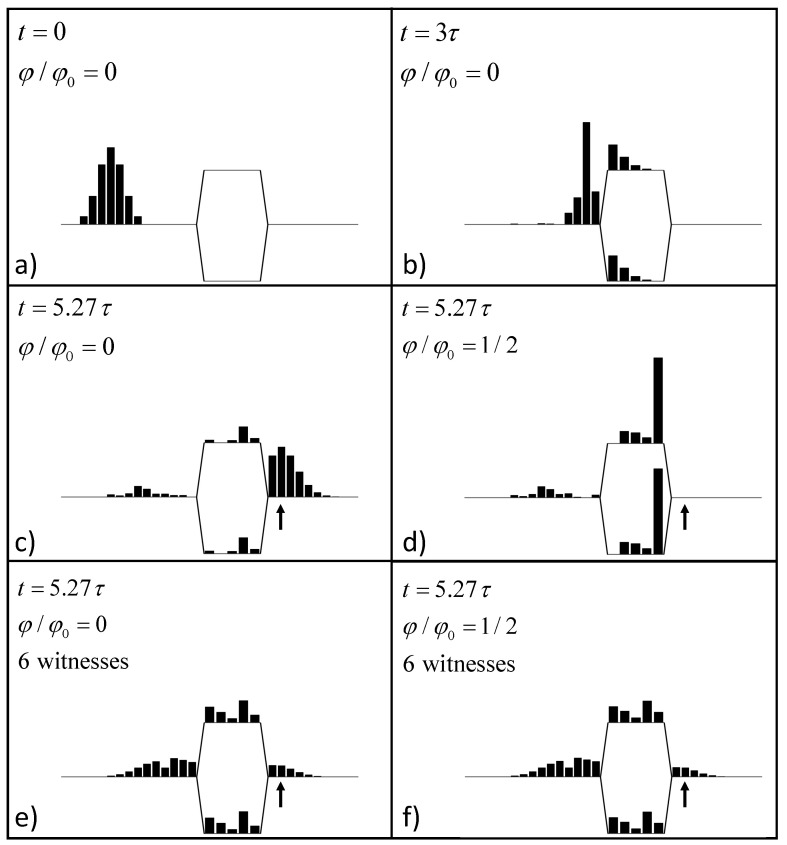
Snapshots of the probability distribution. The probability at each site shown in Figure 2 is represented by the height of the solid bar at that site. Times are represented in terms of the characteristic time τ=πℏ/γ, where γ is the tunneling matrix elements between sites. (**a**) The initial state with the incoming wave packet described by Equation (5). The magnetic flux is zero. (**b**) The probability distribution at t=3τ. Some reflection from the y-branch on the left is evident. (**c**) At $t=T_{f}\equiv 5.27\tau$, the wave packet has emerged into the output lead. The arrow indicates the output site $j_{out}$. For zero magnetic flux, the wavefunction from top and bottom branches interfere constructively. (**d**) The same situation as panel (**c**), but with magnetic flux $\varphi /\varphi_{0}=1/2$. The phase accumulated traversing the top and branches is exactly opposite, leading to completely destructive quantum interference and zero output. (**e**) The zero-field case analogous to panel (**c**), but with six minimal witnesses. The witnesses are coupled in the geometry shown in Figure 2 with coupling energy $E_{int}/\gamma =5$. (**f**) The six-witness case with $\varphi /\varphi_{0}=1/2$ analogous to panel (**d**). The presence of the witnesses in panels (**e**,**f**) dramatically reduces the coherent quantum interference evident in panels (**c**,**d**).

Consider first the case where $\varphi /\varphi_{0}=0$, i.e., no applied field. Figure 3a shows the probability distribution at t=0 for the wave packet given by (5). The input lead is long enough to assure that the initial state has no amplitude in the two branches. A snapshot of the probability density at t=3τ is shown in Figure 3b. The left y-branch point that connects top and bottom leads causes some scattering back to the input. Such branching must cause reflections as noted in [20].

Figure 3c shows the probability distribution at $t=T_{f}\equiv 5.27\tau$. This time is chosen so that the peak of the wave packet is at the second site from the left in the output lead (index j=27). We denote the basis state for this site $|j_{out}\rangle$, and take the signal output to be the probability for the electron to be found at this site at $T_{f}$.

(6) $$P_{out}={\left|\langle j_{out}\;|\;\psi (T_{f})\rangle \right|}^{2}$$

The output site is indicated by the vertical arrow in Figure 2 and Figure 3c–f. The remainder of the output lead is long enough that the effects of reflection from the right end is negligible. Note that in Figure 3c, some probability density has backscattered from the right y-branch back into both top and bottom branches.

When $\varphi /\varphi_{0}$ is zero or any integer, the result is identical to that shown in Figure 3c—there is constructive interference at the output. When $\varphi /\varphi_{0}=1/2$, or any odd half integer, interference between the two paths is completely destructive and the output is zero, as shown in Figure 3d.

**Figure 4:**
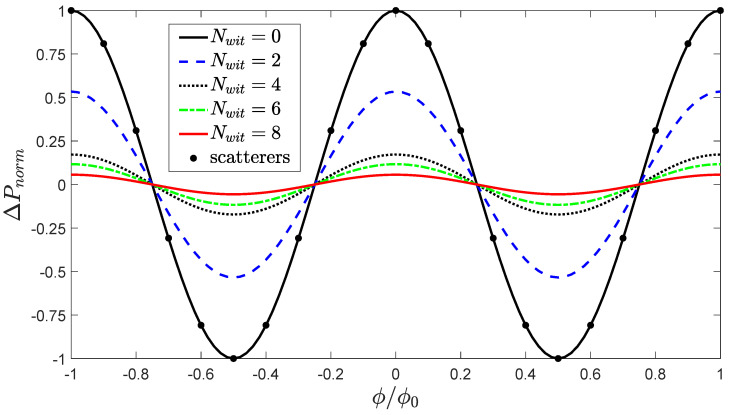
Quantum interference in the presence of blind witnesses. The values of the normalized output probability (Equations (6) and (7)) are shown as a function of the magnetic flux for different numbers of witnesses. The visibility V of the normalized interference pattern is half the peak-to-peak value. The interaction energy between the device and each witness is $E_{int}=5\gamma$. The solid circles are for the case when there are no witnesses, but instead fixed scatterers at the $\left[1,1^{\prime },3,3^{\prime },5,5^{\prime }\right]$ sites. The values fall exactly on the curve for zero witnesses.

The solid (black) line in Figure 4 shows the normalized output $\Delta P_{norm}$ as a function of magnetic flux through the loop $\varphi /\varphi_{0}$. Let $P_{max}$ and $P_{min}$ be the maximum and minimum value of $P_{out}\left(\varphi \right)$, respectively. The midpoint value is then $P_{mid}=\left(P_{max}+P_{min}\right)/2$ and the normalized output is

(7) $$\Delta P_{norm}=\frac{P_{out}-P_{mid}}{P_{mid}}$$

Figure 4 shows that $P_{norm}$ is periodic in $\varphi /\varphi_{0}$ with maximal interference fringes that extend from −1 to +1. The interference visibility, defined by

(8) $$V=\frac{P_{max}-P_{min}}{P_{max}+P_{min}},$$

is half the peak-to-peak value of $P_{norm}$ and in this case is equal to 1.

## 4. Dynamics with Witnesses

Our approach here is not to perform a measurement on either one or both paths, but to let the particle in each path interact with $N_{wit}$ witnesses that we describe quantum mechanically as part of the same overall system. We model each witness as a quantum double dot that is Coulombically coupled to the device system, as shown schematically in Figure 2. The two basis states of the $m^{th}$ witness are $|\alpha_{m}\rangle$ and $|\beta_{m}\rangle$, representing the states with the particle localized completely on one or the other dot. The α state always denotes the dot that is closest to the device and which is field-coupled to the nearest device site. The quantum state of the $m^{th}$ witness can be written as a superposition of these basis states:

(9) $$|\phi_{w}^{\left(m\right)}\left(t\right)\rangle =a_{m}\left(t\right)|\alpha_{m}\rangle +b_{m}\left(t\right)|\beta_{m}\rangle .$$

We choose the zero of energy so the onsite energy for each dot is zero, and therefore write the Hamiltonian for each witness in isolation as simply

(10) $${\hat{H}}_{w}^{\left(m\right)}=-\gamma_{w}\left(|\alpha_{m}\rangle \langle \beta_{m}|+|\beta_{m}\rangle \langle \alpha_{m}|\right).$$

In the absence of interactions between the device and the witnesses, the Hamiltonian for the combined system can be written as

(11) $$\hat{H_{c}}=\sum_{m}{\hat{H}}_{w}^{\left(m\right)}+{\hat{H}}_{d}.$$

The quantum state of the combined system is the direct product of the individual witness states and the device state:

(12) $$|\Psi \rangle =|\phi_{w}^{\left(1\right)}\rangle ⊗|\phi_{w}^{\left(2\right)}\rangle \cdots ⊗|\phi_{w}^{\left(N_{wit}\right)}\rangle ⊗|\psi \rangle$$

The Hamiltonian representing the interaction between a charge on the α dot of the $m^{th}$ witness and a charge on the nearest device site *j* is

(13) $${\hat{H}}_{int}^{\left(m,j\right)}=E_{int}\;{\hat{I}}_{w}^{\left(1\right)}⊗{\hat{I}}_{w}^{\left(2\right)}\cdots ⊗\left(|\alpha_{m}\rangle \langle \alpha_{m}|\right)\cdots ⊗{\hat{I}}_{w}^{\left(N_{wit}\right)}⊗\left(|j\rangle \langle j|\right).$$

Here, $E_{int}$ is the interaction energy and ${\hat{I}}_{w}^{\left(m\right)}$ is the identity operator for the $m^{th}$ witness state. There is no tunneling between device sites and witnesses; the interaction is purely Coulombic. Consider, for example, the witness shown at the left edge of the top branch in Figure 2 at the position labeled 1. The interaction couples the first witness (m=1) of six witnesses to the nearby device site (in this case, the site with index j=16).

A simple classical picture of the function of the witness as an electrometer that could receive which-path information is as follows. Suppose an electron is moving through one branch of the device and is momentarily resident on the $j^{th}$ site. If at the same time the witness charge is on the nearby $\alpha_{m}$ dot, then there is an increase of $E_{int}$ in the energy of the system because of the Coulomb interaction. The witness charge would then be pushed off the α dot onto the β dot and a measurement of the occupancy of either would reveal which path through the device the electron had taken.

The complete Hamiltonian is composed of the combined device and witness Hamiltonian (without interactions) and the interaction term for each pair consisting of a witness *m* and its associated device site *j*:

(14) $$\hat{H}=\hat{H_{c}}+\sum_{pairs\;m,j}{\hat{H}}_{int}^{\left(m,j\right)}$$

The time evolution of $|\Psi \rangle$ is calculated directly using the full Hamiltonian:

(15) $$|\Psi (t)\rangle =e^{-i\frac{\hat{H}}{\hbar }t}|\Psi (0)\rangle .$$

To calculate the probability density at each device site *j*, we embed corresponding projection operator in the larger Hilbert space that describes the whole system.

(16) $${\hat{P}}_{j}={\hat{I}}_{w}^{\left(1\right)}⊗{\hat{I}}_{w}^{\left(2\right)}\cdots ⊗{\hat{I}}_{w}^{\left(N_{wit}\right)}⊗\left(|j\rangle \langle j|\right)$$

The probability of finding the particle at device site *j* is then

(17) $$P_{j}\left(t\right)=\langle \Psi (t)\;|\;{\hat{P}}_{j}\;|\;\Psi (t)\rangle .$$

Now, we add the further (blind witness) restriction that the tunneling energy is $\gamma_{w}=0$. This means that the witnesses have no internal dynamics—all the matrix elements of ${\hat{H}}_{w}$ in the $\left\{|\alpha \rangle ,|\beta \rangle \right\}$ basis are zero. The occupancy of the α and β sites cannot change, so $|a_{m}{|}^{2}={\left|b_{m}\right|}^{2}=1/2$ in Equation (9). This does not mean that the witnesses have no quantum dynamics; they entangle with the device system through (13) and there are quantum mechanical degrees of freedom associated with the phases of $a_{m}$ and $b_{m}$. The entangled system, of course, cannot in general be factored into quantum states of the witnesses and the quantum states of the device.

The Hamiltonian of the system is determined by the number of witnesses $N_{wit}$, the positions where each witness is attached to the device, the hopping energy γ (which sets the time scale τ of the motion), the strength of the witness–device interaction $H_{int}/\gamma$, and the magnetic flux through the loop $\varphi /\varphi_{0}$.

We solve for the time evolution of the quantum state with the incoming wave packet given by the initial device state (5), as in Section 3, but now in the presence of witnesses symmetrically attached to top and bottom branches. The initial state of each witness is taken to be the symmetric state:

(18) $$|\phi_{w}^{\left(m\right)}\left(0\right)\rangle =\left(|\alpha_{m}\rangle +|\beta_{m}\rangle \right)/\sqrt{2}.$$

Figure 3e,f shows snapshots of the probability for the same time $T_{f}$ as are shown in Figure 3c,d, but now in the presence of six witnesses in the top and bottom branches. The witnesses are at positions $\left[1,1^{\prime },3,3^{\prime },5,5^{\prime }\right]$, as shown in Figure 2, and $E_{int}/\gamma =5$. Figure 3e shows the snapshot when $\varphi /\varphi_{0}=0$, and Figure 3f shows $\varphi /\varphi_{0}=1/2$.

In contrast with Figure 3c,d, the probabilities shown in Figure 3e,f are very similar (though not identical). The presence of minimal witnesses has quenched both constructive interference for $\varphi /\varphi_{0}=0$ and destructive interference for $\varphi /\varphi_{0}=1/2$.

Each witness causes reflection of the wave packet in the device. The probability density in the branches is the result of multiple reflections from both the y-branches on either end and from the witnesses. For Figure 3e,f, however, the probability distribution in the top branch is identical to the distribution in the bottom branch of the device; the symmetry is preserved.

Figure 4 shows the normalized output probability for magnetic flux $\varphi /\varphi_{0}\in \left[-1,1\right]$ for different numbers of witnesses. For the case of two witnesses, they are at the $\left[3,3^{\prime }\right]$ positions shown in Figure 2. For the 4-witness case, the witnesses are positioned at $\left[1,1^{\prime },5,5^{\prime }\right]$; for the 6-witness case, the witnesses are positioned at $\left[1,1^{\prime },3,3^{\prime },5,5^{\prime }\right]$; and for eight witnesses the positions are $\left[1,1^{\prime },2,2^{\prime },4,4^{\prime },5,5^{\prime }\right]$. As the number of witnesses increases, the interference visibility V is quenched. It is remarkable that with only eight witnesses, this fundamental quantum interference is so strongly reduced. Insofar as the witnesses can be thought of as representing the effect the environment, a very minimal environment is effective at suppressing interference. It should be emphasized that the whole system remains coherent, though the entropy of the device and of each witness increases, as we will see in the next section.

**Figure 5:**
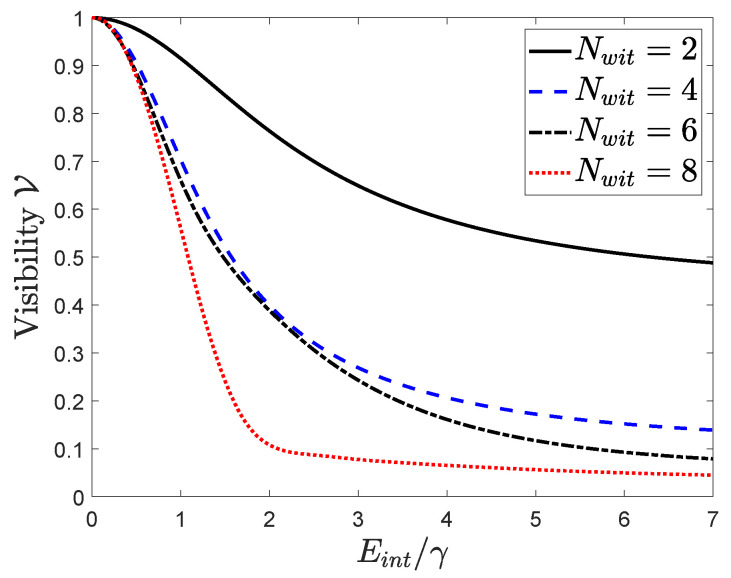
Visibility of quantum interference. The visibility given by Equation (8) is plotted as a function of the interaction strength between the device and the witnesses $E_{int}$ for different numbers of minimal witnesses.

Figure 5 shows the visibility of the interference as a function of $E_{int}/\gamma$, the scaled interaction energy between the device, and the witnesses. The figure shows the result for two, four, six, and eight witnesses, always symmetrically placed in the top and bottom branches. Increasing the strength of the interaction decreases the visibility, though not without limit. For the six-witness case, for example, the visibility at $E_{int}/\gamma =5$ is 11.7%. For $E_{int}/\gamma =50$, visibility decreases to 4.8%, and is essentially the same for $E_{int}/\gamma =500$ (not shown).

Because the witnesses necessarily cause scattering in the branches, one might wonder if the scattering by itself is the source of the observed decoherence. The solid dots in Figure 4 show the output when the witnesses are removed and replaced with fixed potential scatterers. We add a scattering term ${\hat{H}}_{s}$ to the device Hamiltonian of Equation (1):

(19) $$\hat{H}={\hat{H}}_{d}+\sum_{k_{s}}|k_{s}\rangle V_{s}\langle k_{s}|,$$

where $V_{s}=5\gamma$, and the index $k_{s}$ runs over the $\left[1,1^{\prime },3,3^{\prime },5,5^{\prime }\right]$ sites. The resulting interference pattern has visibility V=1; the interference pattern is the same as that of the no-witness case. Scattering alone does not quench quantum interference—it takes the presence of witnesses.

## 5. Dynamics of Witnesses

The classical degrees of freedom of each witness are frozen because $\gamma_{w}=0$. Dot occupancy cannot change, but the quantum degrees of freedom are affected by the passage of the device electron. For each witness *m*, we define the coherence operators ${\hat{\lambda }}_{x}^{\left(m\right)}$, ${\hat{\lambda }}_{y}^{\left(m\right)}$, and ${\hat{\lambda }}_{z}^{\left(m\right)}$ of the two-state witness system in the full system by embedding the Pauli operators for the $m^{th}$ witness, ${\hat{\sigma }}_{x}^{\left(m\right)}$, ${\hat{\sigma }}_{y}^{\left(m\right)}$, and ${\hat{\sigma }}_{z}^{\left(m\right)}$, in the larger Hilbert space.

(20) $$\begin{array}{cc} {\hat{\lambda }}_{x}^{\left(m\right)} & ={\hat{I}}_{w}^{\left(1\right)}⊗{\hat{I}}_{w}^{\left(2\right)}\cdots ⊗{\hat{\sigma }}_{x}^{\left(m\right)}\cdots ⊗{\hat{I}}_{w}^{\left(N_{wit}\right)}⊗{\hat{I}}_{D}\\ {\hat{\lambda }}_{y}^{\left(m\right)} & ={\hat{I}}_{w}^{\left(1\right)}⊗{\hat{I}}_{w}^{\left(2\right)}\cdots ⊗{\hat{\sigma }}_{y}^{\left(m\right)}\cdots ⊗{\hat{I}}_{w}^{\left(N_{wit}\right)}⊗{\hat{I}}_{D}\\ {\hat{\lambda }}_{z}^{\left(m\right)} & ={\hat{I}}_{w}^{\left(1\right)}⊗{\hat{I}}_{w}^{\left(2\right)}\cdots ⊗{\hat{\sigma }}_{z}^{\left(m\right)}\cdots ⊗{\hat{I}}_{w}^{\left(N_{wit}\right)}⊗{\hat{I}}_{D} \end{array}$$

We can then calculate the components of the coherence (Bloch) vector $\vec{\lambda }$ for each witness *m*.

(21) $$\lambda_{x}^{\left(m\right)}=\langle \Psi \;|\;{\hat{\lambda }}_{x}^{m}\;|\;\Psi \rangle ,\;\lambda_{y}^{\left(m\right)}=\langle \Psi \;|\;{\hat{\lambda }}_{y}^{m}\;|\;\Psi \rangle ,\;\lambda_{z}^{\left(m\right)}=\langle \Psi \;|\;{\hat{\lambda }}_{z}^{m}\;|\;\Psi \rangle$$

For the initial witness states given by (18), $\langle {\hat{\lambda }}_{z}^{\left(m\right)}\rangle =0$, and the lack of tunneling between witness sites assures that it will remain zero at all subsequent times.

From $\lambda_{x}^{\left(m\right)}$ and $\lambda_{y}^{\left(y\right)})$, we can construct the 2×2 reduced density matrix for each witness:

(22) $$\rho^{\left(m\right)}=\frac{1}{2}\left(1+\lambda_{x}^{\left(m\right)}\sigma_{x}+\lambda_{y}^{\left(m\right)}\sigma_{y}\right).$$

The local state of each witness can be completely characterized by the *x* and *y* components of the coherence vector. It is helpful to recast the information contained in these two real parameters in another form. We define the coherence angle $\theta_{m}$ as the angle the coherence vector of the $m^{th}$ witness makes with the x-axis.

(23) $$\theta_{m}=\arctan\left(\lambda_{y}^{\left(m\right)}/\lambda_{x}^{\left(m\right)}\right).$$

From the density matrix (22) we can also calculate the von Neumann entropy (in bits) for the $m^{th}$ witness.

(24) $$S_{m}=-\mathrm{Tr}\left(\rho^{\left(m\right)}\log_{2}\left(\rho^{\left(m\right)}\right)\right)$$

The von Neumann entropy $S_{m}$ represents the number of bits of missing local information about the quantum state of witness *m* due to its entanglement with the device system, and through that, to other witnesses [21].

**Figure 6:**
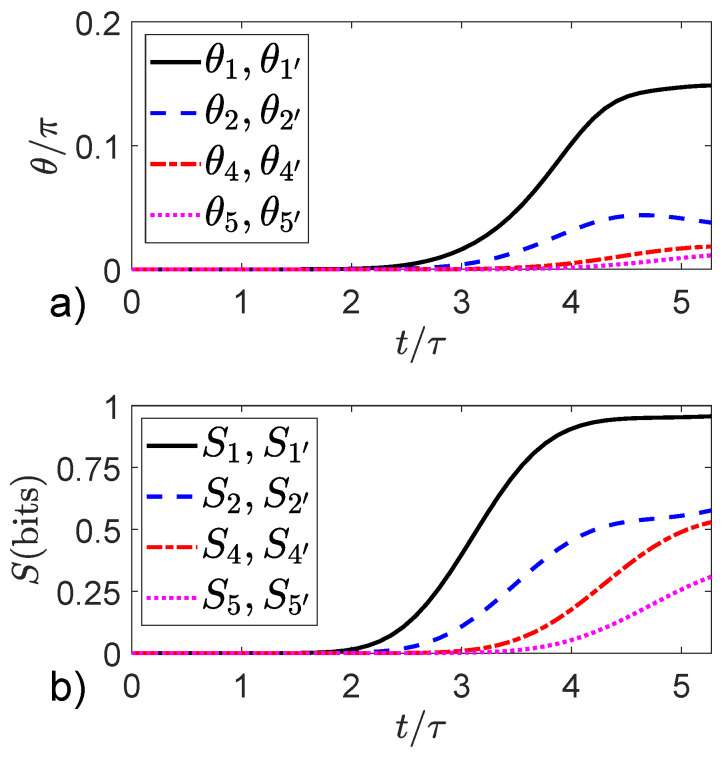
Phase angle and entropy of witnesses. The quantum state of each witness is characterized by its phase angle θ and its von Neumann entropy *S*. The magnetic flux is $\varphi /\varphi_{0}=1/2$. (**a**) The phase angle as a function of time up to $T_{f}$ for 8 witnesses. Each curve is labeled with the witness position shown in Figure 2. (**b**) The corresponding witness von Neumann entropy as a function of time. Importantly, the quantum state of symmetrically placed witnesses (e.g., 1 and $1^{\prime }$) are exactly identical and so do not contain information about which path was taken by the device electron.

Figure 6a shows the time development of the coherence angles for the device with eight witnesses, $E_{int}/\gamma =5$, and $\varphi /\varphi_{0}=1/2$. The curves are labeled with the positions of the witnesses shown in Figure 2. The dynamics is calculated from Equation (15), which yields the global (device plus witnesses) system state $|\Psi (t)\rangle$ and localized to particular witnesses through (21). Figure 6b shows the entropy $S_{m}$ for each witness as a function of time. As the wave packet passes by each witness, the coherence angle shifts slightly and the entropy increases due to entanglement with the device. Corresponding plots for the $\varphi /\varphi_{0}=0$ case are nearly identical to those shown in Figure 6. The entropy of the device itself at $t=T_{f}$ is approximately 2.5 bits. One could say that the information missing from the subsystems (device and witnesses) is now in the quantum correlations between them, but that merely restates the observation that the information is present in neither subsystem yet the global state $|\Psi (t)\rangle$ maintains zero entropy.

The time scale shown in Figure 6 goes to $T_{f}$, when the peak of the packet enters the output lead and we consider the interference “experiment” complete. If the time is extended beyond that, the wave packet bounces back and forth from the ends of the device (beyond the model’s primary intent) and the entropy of each witness approaches its maximal value of 1 bit. The extended time for interaction removes nearly all local quantum information from the witnesses, and the best local description becomes a purely classical mixture of the two dot occupancies each with probability 1/2. Figure 6b shows the beginning of that process. The entropy of the device itself also increases to ~3.9 bits in the long run. The maximum possible entropy for the device would be $S=\log_{2}\left(N\right)\approx 5.13$ bits.

The most important feature to note in Figure 6 is that the symmetrically placed witnesses (e.g., those at positions 1 and $1^{\prime }$ or 2 and $2^{\prime }$) are always in exactly identical states. They have the same values of $S_{m}$ and $\theta_{m}$ (or equivalently $\lambda_{x}^{\left(m\right)}$ and $\lambda_{y}^{\left(y\right)}$) at all times. This is true at for φ=0 as well. The witness states bear no asymmetric imprint representing which-path information. The output interference pattern is destroyed even though the witnesses are unable to record which path is taken. Merely the fact of entanglement between witnesses and device is sufficient to destroy the coherent oscillations, as Figure 4 and Figure 5 demonstrate, despite the complete symmetry of the device and the witness states.

## 6. Discussion 

Entanglement with a random environment, as illustrated in Figure 1b, certainly can cause loss of coherence. If, for example, the environment consists of multiple elements whose interaction energy with the target system has a statistical spread, then the phase relationship of different components may average out. This has been shown in a spin system by Cucchietti et al. [22], and we have seen similar effects in a double-dot system with precisely the same blind witnesses as are employed here [23]. No doubt many physical environments have exactly this character. In such cases, there is also a transfer of information from the system to the environment, even if only to the quantum phases of witnesses. The fingerprint of the system state may be recorded in the details of the quantum state of the environment. However, randomness, by design, plays no role in the calculation described here. The witnesses are geometrically regular, the interaction strengths between each witness and the device are identical, and the initial states of the witnesses are all the same. (Varying the initial witness phase angles $\theta_{m}$ has no effect on interference visibility.)

One might wonder if the loss of coherence seen here could be due to the interaction between the multiple spatial wavelengths (momenta) present in the incoming wave packet and the reflections caused by the witnesses and the y-branches. The finite spread of momenta and energies could be thought to average out the interference. However, if that were the case, we would expect to see interference similarly reduced when witnesses are replaced by static potential scatterers. As discussed above and shown in Figure 4 (points on the solid $N_{wit}=0$ line), no reduction in visibility is caused by the presence of scatterers in the branches.

**Figure 7:**
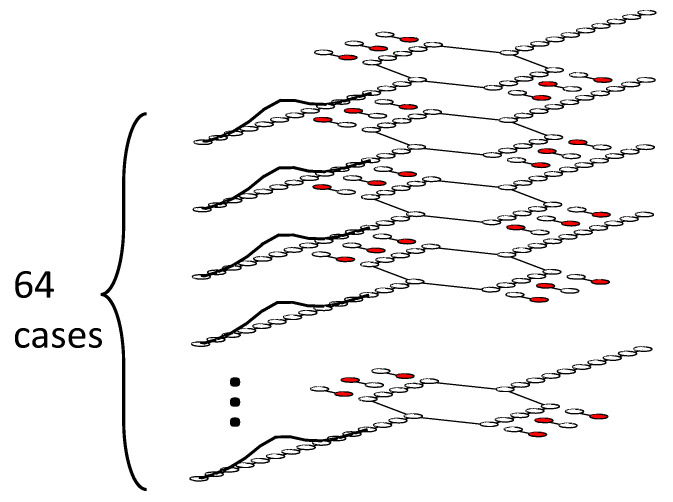
Schematic of the enlarged Hilbert space. For the six-witness case shown, the relevant Hilbert space includes the states of the interference device and all $64=2^{6}$ possible localized states of the witnesses. The initial state is shown here factored into a stack of replicas of the device with different witness configurations. The Hamiltonian does not couple the two localized witness states $|\alpha_{m}\rangle$ and $|\beta_{m}\rangle$, so unitary time evolution of each layer in the stack is independent. The probability amplitude of the initial incoming wave packet is equally distributed among the layers. For each layer, the particular configuration of localized witness states produces a different pattern of reflections in the device. Importantly, the symmetry between witness states in the top and bottom branches is broken for most layers, so the patterns of reflection are different. The amplitudes for each site add coherently to form the overall wavefunction. The complex cancellations of phase between these different possible paths through the Hilbert space results in the quenching of coherence in the interference pattern seen in Figure 4. This phase cancellation, rather than the transfer of which-path information from the device to the witnesses, is the essential source of decoherence.

The crucial effect of the witnesses, even in this highly symmetric geometry, can be understood by considering the evolution of the global state in the expanded Hilbert space that includes the system and witnesses. For the 6-witness case, for example, one can visualize the possible paths through this space as a stack of $2^{6}=64$ layers with replicas of the device states, as shown schematically in Figure 7. Each replica has one of the configurations of the six witnesses in different fully polarized states with either $|\phi_{w}^{\left(m\right)}\rangle =|\alpha_{m}\rangle$ or $|\phi_{w}^{\left(m\right)}\rangle =|\beta_{m}\rangle$. The direct product in the initial state given by Equation (12) generates all the $2^{N_{wit}}$ possible combinations of polarized witness states. The figure schematically shows the initial state of the whole system with the input wave packet distributed equally among the layers. Because the Hamiltonian does not connect different witness states ($\gamma_{w}=0$), each layer evolves independently. For most of these layers (all but 8), the witness configurations on top and bottom branches are not the same—the symmetry is broken.

Under unitary time evolution, each layer has a different combination of reflections from the specific configuration of witnesses in that layer, with different amplitudes and complex phases as a result. Although the occupancy of witness dots in each layer cannot change, each witness has a phase degree of freedom which becomes entangled as the injected wave packet moves by. The probability at the output is obtained by summing the amplitudes from all the layers and taking the absolute square of the result. The partial cancellations from different phase factors in each layer weakens the constructive or destructive interference at the output. The variety of paths through the Hilbert space, most of which have a broken symmetry between top and bottom branches, results in the degradation of the coherent interference pattern at the output. The reason increasing the number of witnesses is so effective at suppressing the interference visibility is that the number of paths (layers in Figure 7) increases exponentially with the number of witnesses.

This analysis is consistent with Zurek’s decoherence and the einselection paradigm [24]. Elsewhere, we have seen the clear emergence of pointer states in an environment of randomly positioned and oriented minimal double-dots, just as are used here, and the resultant quenching of Rabi oscillations, another quintessential quantum effect [23]. We have similarly seen that an environment of random blind double-dot witnesses are sufficient to produce the loss of two-particle entanglement in a system undergoing unitary time evolution [25]. Most environments can indeed receive an imprint from the system sufficient to count as an information transfer from system to environment, and the multiplicity of those records favors system pointer states.

The present calculation shows that the creation of an environmental record of the system’s which-path information is not necessary for quenching the interference pattern. The essential feature is simply entanglement, the system becomes embedded in the dynamics of the much larger Hilbert space that includes all the witnesses. Even in a situation with maximum overall symmetry of device and witnesses, the individual dynamical paths through the larger space need not retain that symmetry. The resultant phase cancellation among the paths destroys coherence.
